# First evidence for STING SNP R293Q being protective regarding obesity-associated cardiovascular disease in age-advanced subjects – a cohort study

**DOI:** 10.1186/s12979-020-00176-y

**Published:** 2020-03-14

**Authors:** Lutz Hamann, Malgorzata Szwed, Malgorzata Mossakowska, Jerzy Chudek, Monika Puzianowska-Kuznicka

**Affiliations:** 1 Institute for Microbiology and Infection Immunology, Charité University Medical Center, CBF, Hindenburgdamm 27, 12203 Berlin, Germany; 2 Department of Human Epigenetics, Mossakowski Medical Research Centre, Polish Academy of Sciences, Warsaw, Poland; 3 PolSenior Project, International Institute of Molecular and Cell Biology, Warsaw, Poland; 4 Department of Internal Medicine and Oncological Chemotherapy, Medical School in Katowice, Medical University of Silesia, Katowice, Poland; 5 Department of Geriatrics and Gerontology, Medical Centre of Postgraduate Education, Warsaw, Poland

**Keywords:** Inflamm-aging, Obesity, STING, Polymorphism

## Abstract

Obesity is a risk factor for several aging-related diseases such as type 2 diabetes, cardiovascular disease, and cancer. Especially, cardiovascular disease is triggered by obesity by inducing vascular senescence and chronic low-grade systemic inflammation, also known as *inflamm-aging*. Released molecules from damaged cells and their recognition by the innate immune system is one of the mechanisms driving *inflamm-aging.* Obesity results in mitochondrial damage, leading to endothelial inflammation triggered by cytosolic mtDNA via the cGAS/STING pathway. Recently, we have shown STING SNP R293Q to be associated with a decreased risk for aging-related diseases in current smokers. Since current smoking triggers DNA damage that, similar to obesity, may result in the release of DNA into the cytoplasm, we hypothesized that the cGAS/STING pathway can modify the phenotype of aging also in obese subjects. Therefore, the objective of our study was to investigate whether STING R293Q is associated with aging-related diseases in obese individuals. We indeed show that STING 293Q is associated with protection from combined aging-related diseases (*P* = 0.014) and, in particular, cardiovascular disease in these subjects (*P* = 0.010). Therefore, we provide the first evidence that stratification for obesity may reveal new genetic loci determining the risk for aging-related diseases.

## Background

Aging is a complex process characterized by a continuous loss of physiological integrity, resulting in the onset of aging-related pathologies, e.g. cardiovascular disease (CVD), cancer, type 2 diabetes (DM-T2), chronic lung diseases, and cognitive impairment. Among them, CVD is worldwide the most frequent cause of mortality [1].

Several hallmarks of aging have been proposed by Lopez-Otin et al. [2], including genomic DNA instability, altered intercellular communication, and mitochondrial dysfunction. The DNA damage is known for a long time to be associated with aging-related pathologies [3]. Several premature aging syndromes, such as Hutchinson-Gilford Progeria syndrome, are driven by the enhanced DNA damage, accompanied by the release of genomic DNA into the cytoplasm [4]. An important aspect of altered cellular communication accompanying aging in mammals is *inflamm-aging*, a condition of systemic chronic low-grade inflammation [5]. *Inflamm-aging* is well known to be associated with aging-related diseases [6] and may result from the accumulation of tissue damage, cell-free or cytosolic DNA, and cellular senescence. Senescent cells accumulate with age and secrete inflammatory cytokines by entering the secretory-associated phenotype [5, 7]. Mitochondrial dysfunction and the release of increased amounts of free radicals (free radical theory) have also been supposed to trigger aging [8]. However, also other mechanisms of mitochondrial dysfunction have been discussed to be involved in aging [9]. Plasma levels of free mitochondrial DNA (mtDNA) increases with age and are associated with *inflamm-aging* [10]. Cytoplasmic mtDNA released from dysfunctional mitochondria fuel *inflamm-aging* via innate immune receptors [11].

Obesity is an important risk factor for several aging-related diseases. In particular, CVD and DM-T2 are triggered by obesity-induced chronic low-grade inflammation [12]. For CVD, obesity is one of the most important risk factors [13] and has also been shown to be associated with endothelial senescence [14]. Recently, it has been demonstrated in mice model that free fatty acid diet induces mitochondrial damage, resulting in the release of mtDNA into the cytosol, triggering endothelial inflammation and insulin resistance by activation of the cGAS/STING pathway [15]. Notably, STING is a key innate immune receptor involved in DNA sensing and plays a pivotal role in both innate immune sensing of pathogens and senescence. Innate immune signaling via cGAS/STING promotes senescence through the secretion of inflammatory cytokines and chemokines, triggering the process of *inflamm-aging*. In addition, cGAS, as well as STING KO mice, exhibit a reduced senescence-associated secretory phenotype (SASP) upon irradiation compared to Wt mice [16, 17].

PolSenior is a multicenter, interdisciplinary project designed to assess the health and socio-economic status of Polish Caucasians aged ≥65 years [18]. By genotyping participants of this program, we showed previously that the STING 293Q allele (rs7380824) is associated with protection from aging-related diseases in a subgroup of current smokers, leading to the hypothesis that the decreased sensitivity of innate immune receptors, by lowering the process of *inflamm-aging,* may be associated with healthy aging*.* We postulated that cytoplasmic DNA induced by genotoxic effects of smoking possibly stimulates the process of *inflamm-aging* via STING, which is decreased by the less functional 293Q allele [19]. To analyze whether STING 293Q allele has a similar effect on aging-related diseases in obese subjects we excluded all current smokers and analyzed only sub-cohorts of obese (*N* = 931, body mass index (BMI) ≥30 kg/m^2^) and non-obese (*N* = 1948, BMI < 30 kg/m^2^) subjects. We could show here that this allele is also associated with protection from aging-related diseases, *P* = 0.014, in these subjects. This effect is more pronounced for CVD, *P* = 0.010, and supports our hypothesis of less *inflamm-aging* by mutations in innate immune receptors being protective regarding aging-related diseases.

### Common risk factors for aging-related diseases in the study cohort

First, we analyzed the distribution of common risk factors for aging-related diseases among obese and non-obese subjects. Since we have shown previously that among current smokers STING 293Q is strongly associated with the decrease in risk for aging-related diseases, we excluded all these individuals from the present analysis. As shown in Table 1, age, sex, and former smoking status differed significantly between both groups with *P* < 0.001 for sex and age, and *P* = 0.007 for former smokers. Therefore, subsequent calculations were corrected for age, sex, and former smoking status. Next, we determined the obesity-associated risk for aging-related diseases by logistic regression analysis. As shown in Table 2, obesity is a strong risk factor for combined aging-related diseases (CVD + chronic lung disease + cancer + DM-T2 + cognitive impairment): *P* < 0.001, CVD: *P* = 0.015, chronic lung diseases: *P* = 0.018, and DM-T2: *P* < 0.001. Obesity is well known to be a strong risk factor for CVD and DM-T2 [12], and our results are consistent with this observation. In our cohort, no significant association with cancer and cognitive impairment could be observed.

**Table 1:** Baseline characteristics of obese and non-obese subjects

	BMI < 30 kg/m^2^, *N* = 1948	BMI ≥30 kg/m^2^, *N* = 931	*P*-value
Mean age (SD)	80.3 (8.4)	76.5 (7.7)	**< 0.001**
Male/female	1077/871	367/564	**< 0.001**
Former smoking yes/no	790/1158	329/602	**0.007**

*P*-values were determined by Student’s T-test for mean age and chi^2^-test for sex and former smoking

**Table 2:** Analysis of the obesity-associated risk for aging-related diseases

Diseases (N: cases/controls)	*P*-value	OR (95%CI)
Aging-related diseases (2009/810)*	**< 0.001**	1.595 (1.323–1.925)
Cardiovascular disease (382/810)	**0.015**	1.405 (1.068–1.846)
Chronic lung disease (160/810)	**0.018**	1.579 (1.081–2.307)
Cancer (47/810)	0.362	0.709 (0.338–1.486)
DM-T2 (177/810)	**< 0.001**	2.359 (1.667–3.338)
Cognitive impairment (343/810)	0.181	1.238 (0.905–1.694)

Analysis was done by logistic regression using SPSS Statistic software package (version 20.0 IBM, Munich, Germany) with BMI < 30 kg/m^2^ as reference and BMI ≥30 kg/m^2^ as predictor, and was corrected for age, sex and former smoking. * 60 samples were excluded from the analysis due to the incomplete disease record

### Inflammatory markers in obese subjects

Since obesity is well known to induce systemic chronic low-grade inflammation, we next analyzed the influence of BMI on the steady-state CRP and IL-6 levels. To exclude subjects with current infections, all subjects with a white blood cell count > 10.000/mm^3^ were removed from this analysis. As shown in Table 3, the steady-state CRP level is higher in the obese compared to non-obese subjects, 4.93 μg/ml vs 4.32 μg/ml, *P* = 0.049 and is further increased in class 2 obese subjects (BMI ≥35 kg/m^2^) up to 5.72 μg/ml, *P* = 0.007. The increase in CRP level is in line with the well-established role of chronic inflammation in obesity-induced pathologies [12]. However, in our study group, the steady-state level for IL-6 exhibits only a marginal, non-significant increase.

**Table 3:** CRP and IL-6 baseline levels in obese and non-obese subjects

	Mean CRP (μg/ml)	STD	*P*-value	Mean IL-6 (ng/ml)	STD	*P*-value
BMI < 30 kg/m^2^ (*n* = 1846)*	4.32	8.0		3.28	3.4	
BMI ≥30 kg/m^2^ (*n* = 869)*	4.93	6.6	**0.049**	3.22	2.7	0.632
BMI ≥35 kg/m^2^ (*n* = 236)*	5.72	7.1	**0.007**	3.42	2.6	0.397

*P*-value was determined by Student’s T-test. * For some samples, data on the level of inflammatory markers were not available (*n* = 54). Samples with a white blood cell count above 10,000/mm^3^ were excluded (*n* = 110)

### Effect of STING 293Q allele on aging-related diseases

We next determined the effect of STING 293Q allele on the obesity-associated risk for aging-associated diseases. Healthy controls (*n* = 810) having none of the five aging-related diseases were compared to subjects having one or more diseases. For the analysis of a given disease, subjects with multiple diseases were excluded to rule out trade-off effects. Therefore, patients suffering from one given disease were compared to healthy subjects. We show here for the first time that this allele is associated with a decreased risk for obesity-associated combined aging-related diseases with OR: 0.651 (95% CI: 0.462–0.917) and *P* = 0.014. This effect is even stronger for CVD with OR: 0.490 (95% CI: 0.285–0.841), and *P* = 0.010 (Table 4). The risk for chronic lung disease and DM-T2 exhibits no significant association with STING 293Q, although obesity is a strong risk factor for both diseases (Table 2), indicating the involvement of other patho-mechanisms in these pathologies. Also, the risk for cancer and cognitive impairment showed no association with STING 293Q. No significant associations were found in non-obese subjects.

**Table 4:** Analysis of STING 293Q allele effects on certain aging-related diseases in obese subjects

Diseases (N: cases/controls)	*P*-value	OR (95%CI)
Aging-related diseases (683/231)*	**0.014**	0.651 (0.462–0.917)
CVD (127/231)	**0.010**	0.490 (0.285–0.841)
Chronic lung diseases (57/231)	0.062	0.492 (0.233–1.037)
Cancer (10/231)	0.529	0.601 (0.123–2.927)
DM-T2 (88/231)	0.375	0.776 (0.443–1.359)
Cognitive impairment (92/231)	0.554	0.835 (0.459–1.518)

Analysis was done by logistic regression using SPSS Statistic software package (version 20.0 IBM, Munich, Germany) with STING R/R as reference and combined R/Q and Q/Q genotypes as predictor, and correction for age, sex and former smoking. * 17 samples were excluded from the analysis due to the incomplete disease record

### Influence of STING 293Q allele on inflammatory markers in obese subjects

Since we hypothesize that an impaired innate immune signaling in the elderly may be associated with healthy aging, we analyzed the effect of STING 293Q on CRP and IL-6 steady-state levels in obese subjects. In STING 293Q carriers, we found slightly reduced, however not significantly, steady-state levels of both CRP and IL-6. The mean CRP level is reduced from 5.11 μg/L to 4.41 μg/L, and the mean IL-6 level is reduced from 3.30 ng/L to 2.98 ng/L in allele carriers compared to non-carriers (Table 5). Although not significant, these differences are well in line in with our hypothesis of impaired innate immune signaling being protective regarding aging-related diseases due to the decreased *inflamm-aging*. Furthermore, one may speculate that in chronic settings, even slight differences in pro-inflammatory markers may affect the risk for aging-related diseases.

**Table 5:** CRP and IL-6 baseline levels in obese subjects are slightly influenced by STING 293Q

STING	Mean CRP (mg/l)	STD	*P*-value	Mean IL-6 (ng/L)	STD	*P*-value
R/R	5.11	7.1	0.093	3.30	2.8	0.098
R/Q + Q/Q	4.41	4.6		2.98	2.2	

*P*-values were determined by Student’s T-test. Samples with a white blood cell count above 10,000/mm^3^ were excluded

### Combined effect of STING 293Q in obese subjects and current smokers

Since both conditions, obesity and smoking, may result in an enhanced STING signaling due to the increased cytosolic DNA content, we combined current smokers and obese subjects and analyzed the effect of STING 293Q on aging-related diseases in this subgroup. As shown in Table 6, STING 293Q is significantly associated with protection from aging-related diseases, CVD, and chronic lung diseases with *P* < 0.000, *P* = 0.002, and *P* = 0.023, respectively. For DM-T2 we only found a trend for protection with *P* = 0.095. Of note, in case of cancer and cognitive impairment, this association is far from being significant; however, both OR are below 1, indicating a possible significant association in larger cohorts.

**Table 6:** Analysis of STING 293Q allele effects on certain aging-related diseases in obese subjects and current smokers

Diseases (N: cases/controls)	*P*-value	OR (95%CI)
Aging-related diseases (885/346)	**< 0.000**	0.567 (0.426–0.754)
CVD (157/346)	**0.002**	0.467 (0.291–0.749)
Chronic lung diseases (83/346)	**0.023**	0.495 (0.270–0.907)
Cancer (15/346)	0.397	0.573 (0.158–2.081)
DM-T2 (117/346)	0.095	0.660 (0.405–1.075)
Cognitive impairment (119/346)	0.289	0.758 (0.454–1.265)

Analysis was done by logistic regression using SPSS Statistic software package (version 20.0 IBM, Munich, Germany) with STING R/R as reference and combined R/Q and Q/Q genotypes as predictor and correction for age and sex

## Conclusion

GWAS for complex diseases have revealed a large number of associations; however, the expected heritability could not be explained so far because the effect sizes are almost modest, possibly due to rare variants or not included gene-environment interactions [20]. Stratification for specific lifestyle or environmental conditions may reveal additional important genetic variants. Genetic associations with diseases that rely on specific gene-lifestyle interactions have been described previously. Recently, some new genetic loci that are associated with serum lipids and CVD have been detected by a large meta-analysis only after stratification for smoking status [21]. In addition, one variation on 2q12.1 locus has been shown to be associated with blood pressure only in obese subjects [22]. In concert with these findings, we provide the first evidence that stratification for obesity and smoking may reveal new genetic loci determining the risk for aging-related diseases or CVD. However, our work has several limitations. The first limitation is a relatively low sample number and the inclusion of only one ethnic group. On the other hand, however, this group is well characterized in terms of morbidity and other health-related parameters. Second, determination of cytoplasmic mtDNA as well as circulating DNA and correlation with STING 293Q and aging related diseases would be very interesting. Therefore, further studies are needed to confirm the protective effect of the STING 293Q allele.

**Fig. 1:**
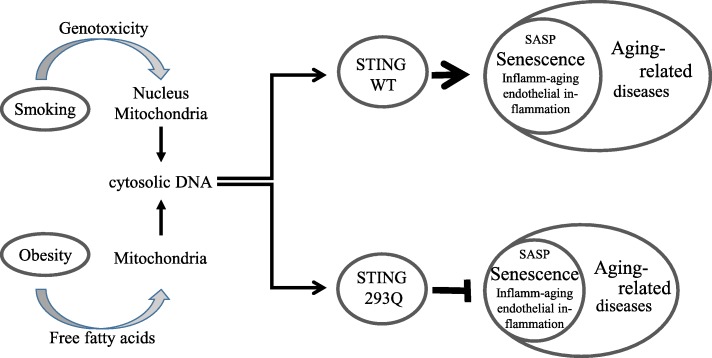
A possible role for STING 293Q in protection from aging-related diseases. Both, smoking and obesity result in the increased DNA damage that in turn results in STING activation and progression of senescence which may be reduced by STING 293Q

The cGAS/STING pathway is well established to be a key regulator of senescence by sensing DNA released upon DNA damage [23]. Cigarette smoke is a genotoxic agent inducing DNA damage [24]. In addition, also obesity induces DNA damage [25] and, furthermore, free fatty acids involved in obesity induce a release of mtDNA into the cytoplasm [15]. Together with our recent observation, we speculate that conditions such as smoking or obesity, resulting in an enhanced release of DNA, may fuel the process of aging in a STING-dependent manner by augmenting senescence, endothelial inflammation, and *inflamm-aging* (Fig. 1). Therefore, STING variants that decrease the sensitivity of the innate immune system towards endogenous DNA possibly reduce the risk for aging-related diseases.

Personalized medicine, including SNP screening, predicting the risk for aging-related diseases, may also stratify for personal environmental or lifestyle factors to reduce the frequency of aging-related diseases. This individualized care of the elderly patient may reduce mortality and health care cost overall.

## Materials and methods

### Study cohort

PolSenior is a multicenter interdisciplinary project designed to assess the health and socio-economic status of Polish Caucasians aged ≥65 years [18]. All participants completed a detailed questionnaire, underwent an examination, including elements of comprehensive geriatric assessment and donated blood for biochemical and genetic analyses. All participants gave a written informed consent for participation in the study. All investigations were carried out in accordance with the ethical guidelines of the 1975 Declaration of Helsinki. The study was approved by the Bioethics Commission of the Medical University of Silesia in Katowice.

### Statistics

Binary logistic regression analyses have been performed employing the IBM SPSS Statistics software package (version 20.0, IBM, Munich, Germany). Sex, age, and smoking status were included as co-factors. Binary variables (sex and smoking) were analyzed by chi^2^-test, continuous variables (age, IL-6, and CRP) were analyzed by Student’s T test.

## Data Availability

Data will be made available on request.
